# IGF2 mRNA binding protein 3 (IMP3) promotes glioma cell migration by enhancing the translation of RELA/p65

**DOI:** 10.18632/oncotarget.17118

**Published:** 2017-04-15

**Authors:** Shruti Bhargava, Abhirami Visvanathan, Vikas Patil, Anuj Kumar, Santosh Kesari, Saumitra Das, Alangar S Hegde, Arimappamagan Arivazhagan, Vani Santosh, Kumaravel Somasundaram

**Affiliations:** ^1^ Department of Microbiology and Cell Biology, Indian Institute of Science, Bangalore, India; ^2^ Department of Translational Neuro-Oncology and Neurotherapeutics, Pacific Neuroscience Institute, John Wayne Cancer Institute, Providence Saint John's Health Center, Santa Monica, California, USA; ^3^ Sri Satya Sai Institute of Higher Medical Sciences, Bangalore, India; ^4^ Departments of Neurosurgery, National Institute of Mental Health and Neuro Sciences, Bangalore, India; ^5^ Departments of Neuropathology, National Institute of Mental Health and Neuro Sciences, Bangalore, India

**Keywords:** IMP3, RNA binding protein, NF-κB signalling, RELA/p65, translation control

## Abstract

The diffusely infiltrative nature of glioblastoma (GBM) makes them highly recurrent. IGF2 mRNA-binding protein 3 (IMP3), a GBM upregulated RNA binding protein, promotes glioma cell migration. An integrative bioinformatics analysis identified p65 (RELA), a subunit of NF-κB heterodimer as a target and an important mediator of IMP3 promoted glioma cell migration. IMP3 increased p65 protein levels without any change in p65 transcript levels, but promoted its polysome association. RIP-PCR demonstrated the binding of IMP3 to p65 transcript. UV crosslinking experiments with *in vitro* transcribed RNA confirmed the specific and direct binding of IMP3 to sites on p65 3′UTR. Further, IMP3 induced luciferase activity from p65 3′UTR reporter carrying wild type sites but not mutated sites. Exogenous overexpression of p65 from a 3′UTR-less construct rescued the reduced migration of glioma cells in IMP3 silenced condition. In addition, IMP3 silencing inhibited glioma stem-like cell maintenance and migration. The exogenous overexpression of 3′UTR-less p65 significantly alleviated the inhibition of neurosphere formation observed in IMP3 silenced glioma stem-like cells. Further, we show that IMP3 is transcriptionally activated by NF-κB pathway indicating the presence of a positive feedback loop between IMP3 and p65. This study establishes p65 as a novel target of IMP3 in increasing glioma cell migration and underscores the significance of IMP3-p65 feedback loop for therapeutic targeting in GBM.

## INTRODUCTION

Glioblastoma (GBM) is one of the leading causes of death caused due to brain tumors. Despite the technical advances in diagnostics and the present treatment modalities the median overall survival of the patients remains 14.6 months [1, 2]. Malignant and heterogeneous nature of the GBM tumors contribute to the observed resistance and recurrence [3]. In the recent past, many authors have demonstrated the existence of a small population of glioma stem-like cells (GSCs) in the tumors which in multiple ways contribute to this heterogeneity and the belligerence of the disease [3–5]. They have been demonstrated to be resistant to the current treatment modalities and are ascertained to be the culprits for the high rates of GBM recurrence [4]. They have also been shown to have high migratory capacity [5]. Thus, the identification of molecules contributing to migratory potential of glioma cells and in GSC maintenance will help in adding lucrative potential targets for adjuvant therapies. Inhibition of such molecules will diminish the tumor load and resistance to existing therapies which may result in lower recurrence rate.

Increasing evidence establishes the role of RNA binding proteins in regulating RNA splicing, stability, localization, modifications and translation [6, 7]. While, recent evidence associates derailed expression or activity of these RBPs with several genetic diseases including cancer, their cellular target repertoire, how they regulate the transcriptome and proteome and whether they can be used for therapeutic intervention warrants further investigation [8–11]. IMP3 is an example of an oncofetal RNA binding protein, which has been associated with various malignancies [12–16]. Few of the bonafide targets of the protein are emerging from the recent research [9]. In this study, we have identified p65 (RelA) as a mediator of IMP3 functions including migration of glioma cell lines and maintenance of GSCs. IMP3 binds to 3′UTR of p65 transcript and enhances its translation. p65 is a subunit of NF-κB transcription factor and this increase in translation of protein also resulted in a higher activity of the NF-κB pathway. Moreover, we demonstrate IMP3 to be a transcriptional target of p65. We have thus, delineated IMP3- NF-κB cascade by which IMP3 contributes in migration of glioma bulk and stem-like cells.

## RESULTS

### Identification of RELA/p65 as an IMP3 target in promoting glioma cell migration

**Figure 1:**
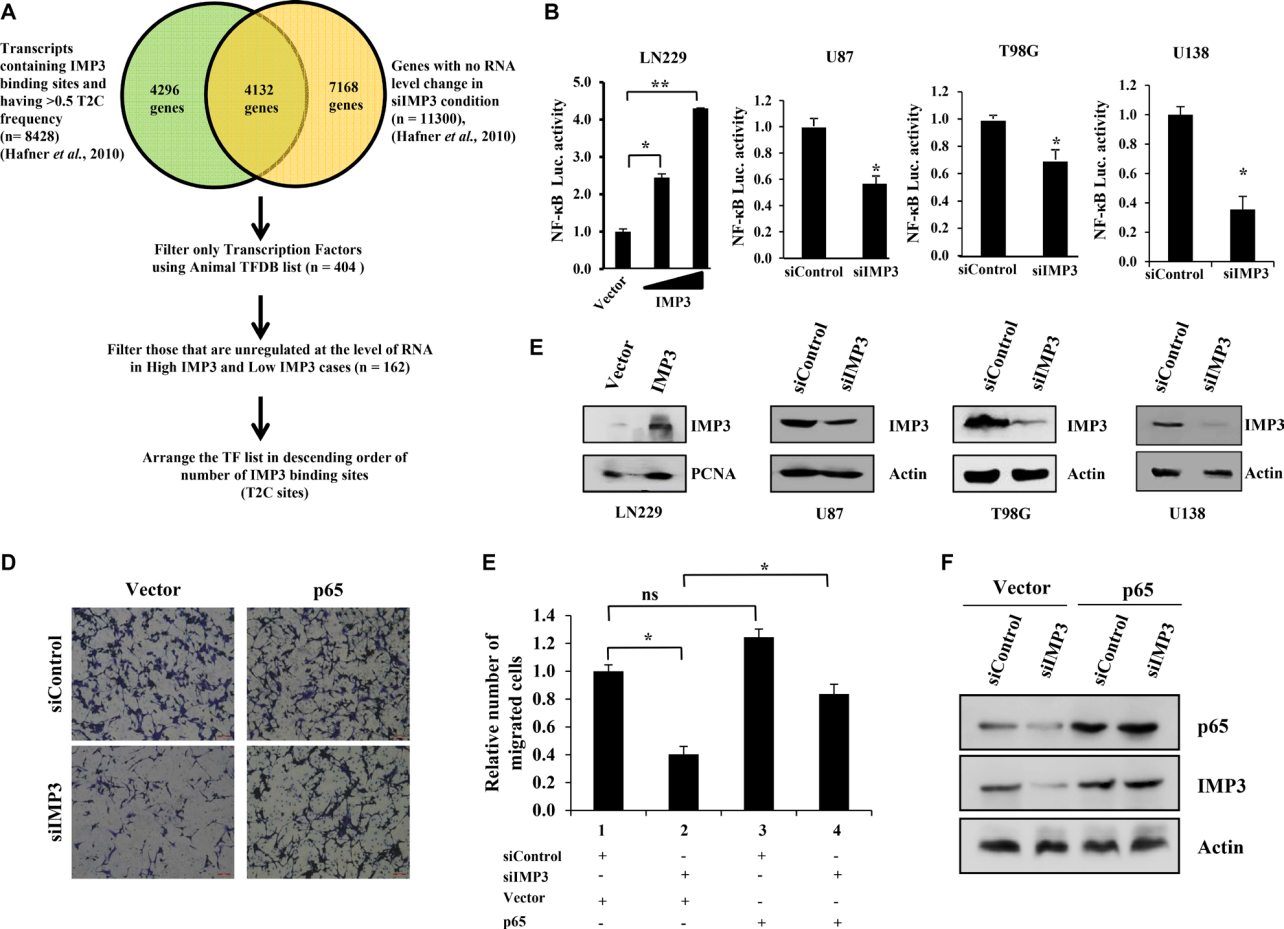
IMP3 expression increases NF-κB activity (**A**) Schematic representation of the strategy employed to find the transcription factor having putative IMP3 binding sites and unregulated at the level of RNA on IMP3 silencing (data provided in GSM545209 and http://www.mirz.unibas.ch/restricted/clipdata/RESULTS/CLIP_microArray/index.html) [19]. (**B**) NF-κB luciferase reporter activity in IMP3 overexpressing LN229 cells and in IMP3 silenced U87, T98G and U138 cells. For overexpression condition, NF-κB dependent reporter luciferase activity was co-transfected with IMP3 overexpression construct. Increasing concentrations of IMP3 expressing vector (1 μg and 2 μg) was used in the assay while keeping the reporter construct constant. Luciferase activity was measured after 48 h of transfection. For IMP3 silenced condition, the readings were taken after 48 h of reporter transfection, while 96 h after siRNA transfection. The activity obtained in vector control conditions was considered to be 1 and relative activities in increasing IMP3 conditions were plotted. (**C**) Western blots showing IMP3 levels upon exogenous IMP3 overexpression (LN229 cells) or silenced conditions (U87, T98G and U138 cells). (**D**) Representative micrographs of migrated U138 cells in the mentioned conditions: siControl with control vector transfection, siControl with p65 overexpression vector transfection, siIMP3 with control vector transfection and siIMP3 with p65 overexpression vector transfection. (**E**) Quantitation of the number of migrated cells is represented as a bar graph. (**F**) Western blots confirming IMP3 silencing and p65 overexpression in U138 cells. For all experiments, a student *t-test* was carried to assign statistical difference in observations made in the conditions indicated. A *p* < 0.05 is represented with *p < 0.01 is represented as ** and *p* < 0.001 is represented as ***.

We have previously demonstrated that high IMP3 GBM tumors are highly migratory/ invasive as seen by the presence of IMP3 positive tumors cells in tumor infiltrating front, perivascular and subpial regions [17]. In this work, we set out to identify IMP3 targets that promote glioma cell migration. An integrated bioinformatics approach was used to identify IMP3 targets that encode transcription factors since they are global regulators (Figure 1A). Many RNA binding proteins regulate their targets at mRNA translation level, and few of the identified IMP3 targets were also regulated at this level [17, 18]. We were particularly keen to identify transcription factors whose expression is regulated by IMP3 at the translation step. To begin with, we utilized the PAR-CLIP data containing a list of mRNAs with IMP3 binding sites and transcriptome data of IMP3 silenced condition in HEK293 cells from Hafner et al. [19]. Firstly, we selected transcripts that contained IMP3 binding sites and whose transcript levels are unaltered under IMP3 silenced conditions (*n* = 4132). Next, this list was compared with transcription factor (TF) data base [20] which identified a list of 404 TFs. We then superimposed the TCGA GBM transcriptome data and short listed 162 TFs with their transcript levels unaltered between high- and low-IMP3 GBM tumors. These TFs were arranged according to the number of IMP3 binding sites present in their mRNA (Supplementary Table 1). Among top 5 genes with 10 or more IMP3 binding sites, we chose p65 (RELA) for further studies since p65 is a member of NF-κB heterodimeric transcription factor complex [21]. NF-κB pathway has been extensively associated with aggressive phenotypes of GBM, especially migration, invasion, angiogenesis, chemo-resistance and GSC maintenance [22–24].

NF-κB primarily exists as a heterodimeric transcription factor consisting of p65 and p50 subunits [21]. To experimentally demonstrate that p65 is a target of IMP3, we measured the p65-dependent luciferase activity in glioma cells after modulating IMP3 levels. Exogenous overexpression of IMP3 in LN229 glioma cells increased luciferase activity in a concentration dependent manner, while knockdown of IMP3 in U87, T98G and U138 cells led to a significant reduction in the activity from NF-κB dependent reporter (Figure 1B and 1C). Next, we assessed the role of p65 as a downstream effector of IMP3 mediated migration. As expected, we observed a reduced migratory capacity of U138 cells upon IMP3 knockdown (Figure 1D and 1E; compare bar 1 with 2). Exogenous overexpression of p65 alleviated the diminished migration observed in IMP3 silenced U138 cells (Figure 1D and 1E; compare bar 4 with 2). Silencing of IMP3 and overexpression of p65 was confirmed using western blot (Figure 1F). Collectively, these results establish p65 as a target of IMP3 in mediating its pro-migratory functions in glioma cells.

### IMP3 activates NF-κB activity by promoting translation of p65 transcript

**Figure 2:**
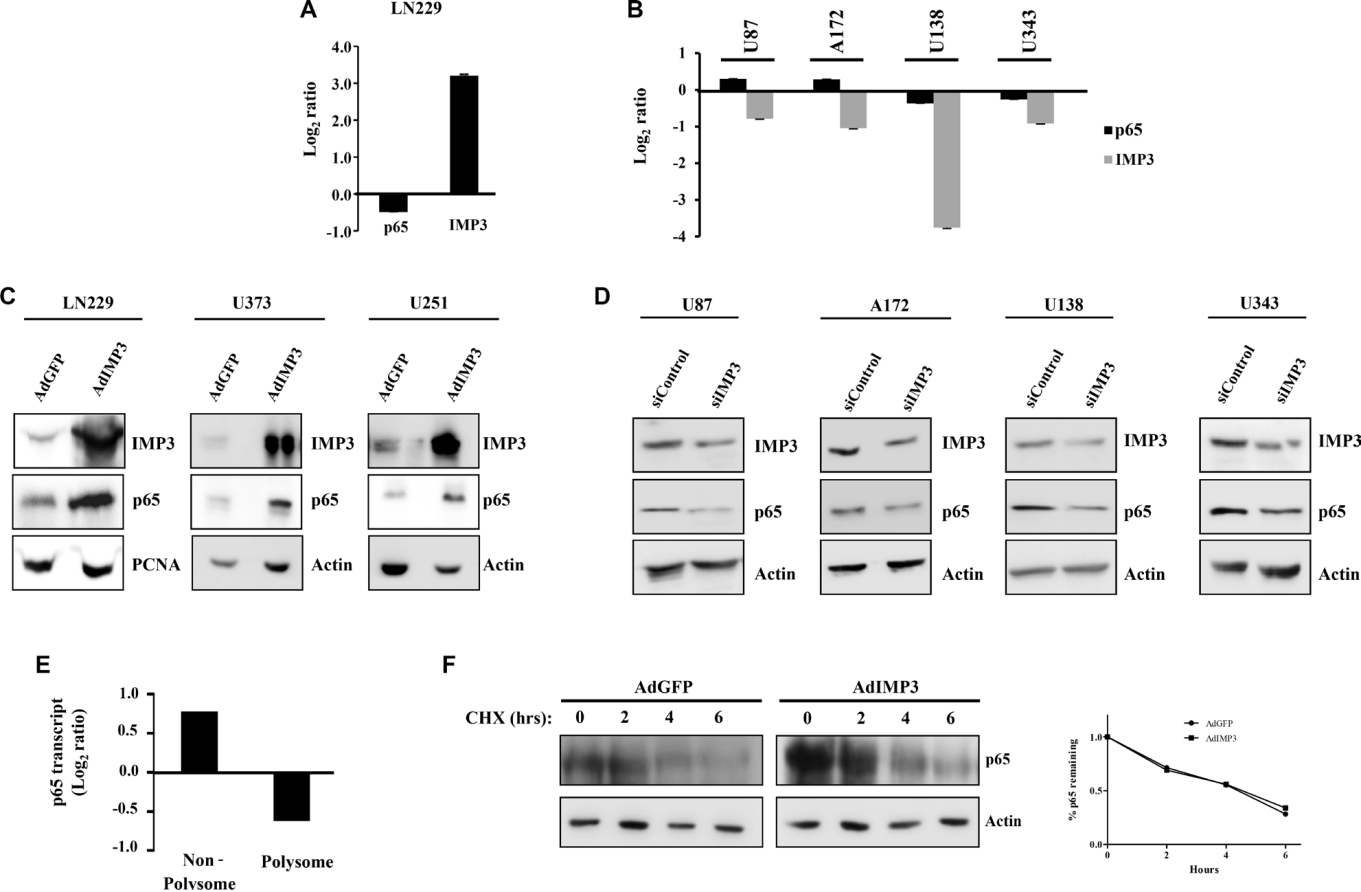
Mechanism of p65 expression regulation by IMP3 (**A**) Transcript levels of p65 and IMP3 in IMP3 overexpressing LN229 cells as assessed by qRT-PCR. Log_2_ ratio of transcript levels in AdIMP3 infected cells relative to AdGFP infected cells was plotted. (**B**) Transcript levels of p65 and IMP3 in IMP3 silenced conditions in glioma cell lines like U87, A172, U138 and U343. Measurement was done using qRT-PCR and the log_2_ ratio of transcript levels in siIMP3 transfected cells relative to siControl transfected cells was plotted. (**C**) Western blotting analysis of lysates from cell lines ectopically overexpressing IMP3 for the levels of IMP3, p65 and loading controls (Actin or PCNA). (**D**) Western blots showing levels of p65 and actin after IMP3 silencing. (**E**) Polysome analysis to measure the changes in p65 transcript translation in IMP3 knockdown condition was performed. p65 transcript levels were measured in non-polysome and polysome fraction in IMP3 and control knockdown condition by qRT-PCR. Log_2_ ratio of transcript levels in siIMP3 transfected cells relative to siControl transfected cells was plotted. (**F**) Western blot analysis followed by cycloheximide chase experiment. p65 levels were measured in these lysates. Quantitation of the remaining p65 expression (at 2, 4 and 6 hours) when compared to 0 hours in the two conditions is shown as the graph (right). For all experiments, a student *t-test* was carried to assign statistical difference in observations made in the conditions indicated. A *p* < 0.05 is represented with *p < 0.01 is represented as ** and *p* < 0.001 is represented as ***.

To dissect the mechanism behind the regulation of p65 and thereby NF-κB activity by IMP3, we checked the p65 transcript and protein levels in IMP3 overexpressed and knockdown conditions. p65 transcript levels in IMP3 overexpressing LN229 cells or IMP3 silenced U87, A172, U138 and U343 cells was found to be unaltered (Figure 2A and 2B). Additionally, there was no significant difference in p65 transcript levels between low IMP3 and high IMP3 GBM samples from TCGA and GSE22866 data sets (Supplementary Figure 1A and 1B). Moreover, we observed no significant correlation between p65 protein and p65 transcript in our patient (GBM) cohort as assessed by immunohistochemical analysis (IHC) and qRT-PCR respectively (*r* = 0.2521, *p* = ns, Supplementary Figure 1C). We next analysed the effect of IMP3 on p65 protein levels. While IMP3 overexpression resulted in many fold increase in p65 protein in LN229, U373 and U251 cells (Figure 2C), IMP3 silencing decreased the p65 protein levels in U87, A172, U138 and U343 cells (Figure 2D). Moreover, we found the protein levels of p65 and IMP3 were significantly correlating in our patient (GBM) cohort as assessed by IHC (*r* = 0.3648, *p* = 0.0175, Supplementary Figure 1D). Furthermore, this increased p65 protein is also functionally active, as evident by nuclear translocation of p65 protein in LN229 and U251 cells with IMP3 overexpression (Supplementary Figure 1E). Conversely, IMP3 knockdown led to reduced nuclear p65 staining in A172 and U343 cells (Supplementary Figure 1F). Thus, IMP3 increases the NF-κB activity by increasing the p65 protein levels, without any change in its transcript level. Further to determine the mechanism behind increased p65 protein levels in the presence of IMP3, we looked at the translation and protein stability of p65. In knockdown conditions of IMP3 in U138 cells, polysome analysis revealed a significant decrease in the p65 transcript level in polysome fraction and a concomitant increase of the transcript in the non-polysome fraction (Figure 2E). To check the effect of IMP3 levels on p65 protein stability, cycloheximide chase experiment was performed. The rate of degradation of p65 protein was seen to be similar in IMP3 overexpression conditions (AdIMP3) and control (AdGFP) LN229 cells, implying that IMP3 may not be playing a role in regulating the protein stability of p65 (Figure 2F). Taken together, IMP3 increases the levels of p65 protein by increasing its mRNA translation.

### IMP3 binds to 3′UTR of p65 RNA

**Figure 3:**
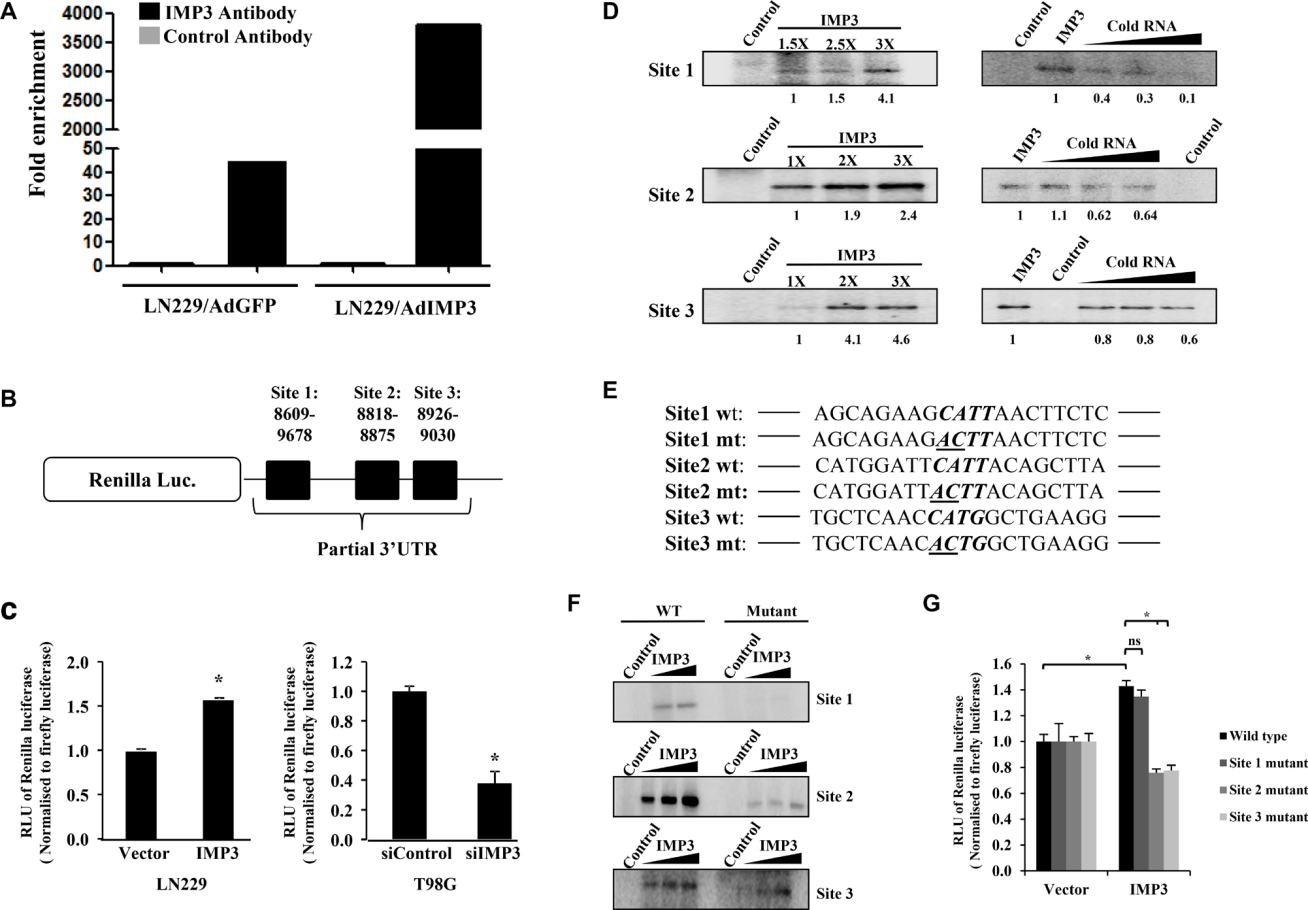
IMP3 binds to p65 transcript (**A**) RNA immuno-precipitation (RIP) followed by qRT-PCR in LN229 cells transduced with AdGFP or AdIMP3 virus. RIP was performed 48 h post infection. p65 transcript levels were measured (qRT-PCR) in control antibody (IgG) or IMP3 antibody immunoprecipitated fractions from GFP virus and IMP3 virus infected cells and depicted. (**B**) Schematic representation of the luciferase construct used, where partial p65 3′UTR containing 3 putative IMP3 binding sites was cloned downstream to Renilla luciferase gene. (**C**) Construct carrying first three putative IMP3 binding sites in p65 3′UTR was transfected in ectopically IMP3 overexpressing cells (LN229) and IMP3 silenced cells (T98G). Luciferase readings were taken 48 h after reporter transfection and relative readings were plotted. (**D**) Phosphorimages after RNA-protein UV crosslinking experiments were performed. α ^32^P-labelled RNAs (~150 bases each) of corresponding sites were incubated in the absence or increasing concentrations of purified IMP3 protein. The radiolabel RNA-protein complexes were resolved on SDS-10% PAGE gels and phosphorimaging was performed (*three left panels*). α ^32^P-labelled RNA was also UV cross-linked to recombinant purified IMP3 protein, with 100, 200 and 400 fold molar excess of unlabelled same site RNA (cold RNA). Images after phosphorimaging for these experiments are also shown (*three right panels*). (**E**) Partial representation of the sites with four nucleotide consensus for IMP3 binding shown in bold letters. The first two mutated bases in the consensus are underlined in the mutated sequence. (**F**) Phosphorimages after RNA UV crosslinking experiments with wild type or the mutant RNA probes for the three sites. (**G**) Luciferase assay performed in IMP3 overexpressing LN229 cells transfected with wild-type or mutated 3′UTR plasmids. Each plasmid only had one site mutated as indicated, with the other two sites having wild type sequence. Luciferase readings were taken after 48 h of reporter plasmid transfection. Relative luciferase readings were calculated and depicted as bar graphs. For all experiments, a student *t-test* was carried to assign statistical difference in observations made in the conditions indicated. A *p* < 0.05 is represented with *p < 0.01 is represented as ** and *p* < 0.001 is represented as ***.

We next sought to investigate whether the increase in p65 translation is mediated *via* direct binding of IMP3 to the p65 transcript. The binding of IMP3 to p65 transcript was confirmed in the RNA immunoprecipitation assays, where the p65 transcripts were found to be enriched in IMP3 overexpressed fraction as compared to control fractions in LN229 cells (Figure 3A). Further analysis of p65 transcript sequence for the presence of IMP3 binding sites revealed that there are 10 potential binding sites, with four of them in 3′ UTR of the transcript (Supplementary Figure 2A). Since IMP3 binding sites are generally enriched in the 3′UTR of transcripts [19], and 3′UTR sites have been implicated in translational regulation, we investigated the importance of four sites present in the p65 3′UTR. Based on the conservation of the sites across the species (Supplementary Figure 2B), the first three sites were taken up for studies.

The activity from a reporter construct with p65 3′UTR having these three sites downstream to Renilla luciferase gene (Figure 3B) is induced in LN229 cells overexpressing IMP3 and is reduced in IMP3 silenced T98G cells (Figure 3C). UV crosslinking experiments using purified IMP3 protein, and radioactively labelled *in vitro* transcribed RNA corresponding to these three sites revealed that all the three sites are capable of binding to IMP3 (Figure 3D, left panel). This binding is specific as the cold RNA of the same site competed efficiently the binding by IMP3 (Figure 3E, right panel). Further, IMP3 failed to bind to a non-specific RNA (Supplementary Figure 2C). The importance of the predicted consensus sequence [19] in these sites for the specific binding by IMP3 was further confirmed by altering the important residues. Conversion of the first two residues CA to AC (Figure 3E) of the four nucleotide consensus 5′-CAUH-3′ [19] significantly abolished the binding by IMP3 (Figure 3F). To test the biological significance of IMP3 binding to p653′UTR, we tested the ability of IMP3 to activate a luciferase reporter construct carrying p65 3′UTR with the mutation in the IMP3 binding consensus as described earlier. To our surprise, site 1 mutation did not significantly affect the ability of IMP3 to activate the reporter activity, while both site 2 or 3 mutations abolished the ability of IMP3 to activate the reporter activity (Figure 3G). These observations underscore the importance of IMP3 binding to site 2 and site 3 over site 1 in physiological conditions. Thus, we conclude from these experiments that IMP3 binds to the p65 3′UTR directly, and this binding may be important for its regulation on p65 expression.

### A positive feedback loop between IMP3 and NF-κB pathway

**Figure 4:**
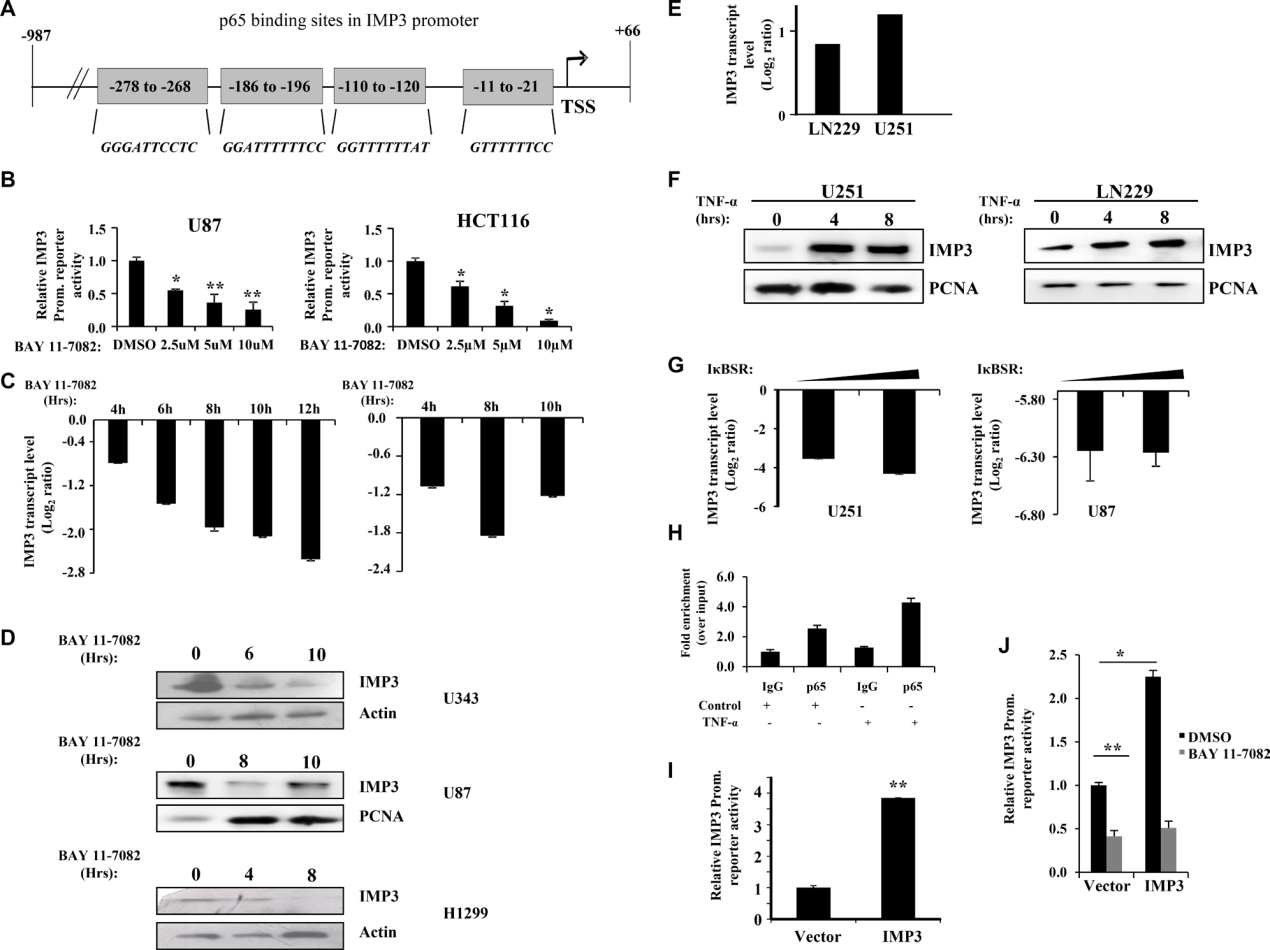
IMP3 gene expression is regulated by NF-κB activity and both participate in a positive feedback loop (**A**) Schematic representation of IMP3 promoter showing putative p65 binding sites revealed by bioinformatics analysis. The positions relative to Transcription Start Site (TSS) are indicated in the boxes. The putative consensus sequence of NF-κB binding in each of the sites is shown in red. (**B**) IMP3 promoter dependent luciferase activity of the cells transfected with IMP3 promoter reporter was measured after treatment with BAY 11-7082 at the indicated concentrations. Luciferase assays were performed 48 h after transfection and 12 h of inhibitor treatment. Luciferase readings were normalized to β-galactosidase readings present in the lysate. (**C**) Measurement of IMP3 transcript levels after treatment of U343 cells (*left panel*) and H1299 cells (*right panel*) with BAY 11-7082 (10 μM) at the indicated time points using qRT-PCR was performed. (**D**) Western blot analysis was performed to measure IMP3 protein levels in lysates of cells treated with BAY 11-7082 (10 μM) for indicated time points. (**E**) Transcript levels of IMP3 in cells treated with TNF-α (10 ng/ml) for 4 h in LN229 and U251 cells, as measured using qRT-PCR. (**F**) IMP3 protein levels in lysates made from cells treated with TNF-α (10 ng/ml) for the indicated time points. (**G**) Transcript levels of IMP3 in U251 and U87 cells after 48 h of IκB super repressor (IκBSR) transfection. Measurements of transcript levels were made using qRT-PCR. (**H**) ChIP was performed in untreated and TNF-α (10 ng/ml) treated U87 cells. qRT-PCR was performed to check the levels of region of IMP3 promoter amplified in p65 and control (IgG) immuno-precipitated chromatin. Relative enrichment of IMP3 promoter region in p65 pull-down over IgG pull-down conditions is shown in TNF-α treated and untreated cells. (**I**) Relative luciferase activity measurements of IMP3 promoter reporter in LN229 cells with ectopic overexpression of IMP3. All readings were recorded after 48 h of reporter transfection. (**J**) Relative luciferase activity measurements of IMP3 promoter reporter in LN229 cells ectopically expressing IMP3 treated or untreated with BAY 11-7082 (10 μM). All readings were recorded after 48 h of reporter transfection and 12 h of inhibitor treatment. For all experiments, a student *t-test* was carried to assign statistical difference in observations made in the conditions indicated. A *p* < 0.05 is represented with *p < 0.01 is represented as ** and *p* < 0.001 is represented as ***.

We then investigated the possibility of IMP3 being transcriptionally activated by NF-κB pathway involving a positive feedback loop. To assess this possibility, we sub-cloned partial region of IMP3 promoter (−987 to +66) having four p65 binding sites upstream to Fire-fly Luciferase gene of pGL3-Basic vector to construct an IMP3 promoter-reporter construct (Figure 4A). Treatment of U87 and HCT116 cells with a pharmacological inhibitor to NF-κB pathway, BAY 11-7082 (an inhibitor of IKK complex activity) resulted in a dose dependent inhibition of luciferase activity from IMP3 promoter-reporter construct (Figure 4B). BAY 11-7082 treatment also resulted in reduced IMP3 transcript (U343 and H1299 cells) and protein levels (U87, U343 and H1299 cells) (Figure 4C and 4D). As expected, under these conditions, BAY 11-7082 treatment inhibited reporter activity from NFκB-dependent reporter (Supplementary Figure 3A). Further, activation of NF-κB pathway by TNF-α led to increase in IMP3 RNA and protein levels in LN229 and U251 cells (Figure 4E and 4F). TNF-α treatment resulted in the activation of reporter activity from NFκB-dependent reporter as anticipated (Supplementary Figure 3B). Additionally, a dominant negative form of IκB (IκBSR), an inhibitor of NF-κB pathway, decreased IMP3 transcript levels in U251 and U87 glioma cells (Figure 4G). We also found a significant positive correlation between p65 protein (as determined by immunohistochemistry) and IMP3 transcript levels (as determined by qRT-PCR) in our cohort of GBM patient samples (*r* = 0.2976, *p* = 0.0304, Supplementary Figure 3C). We went ahead to check if p65 binds directly to IMP3 promoter by performing ChIP-qPCR. We found enhanced p65 occupancy on the IMP3 promoter region both in endogenous and TNF-α activated condition in U87 cells (Figure 4H). Analysis of IMP3 promoter region that was amplified for ChIP-qPCR in UCSC genome browser [25] also showed an enrichment of activated histone marks (H3K27Ac marks) (Supplementary Figure 3D). To investigate whether IMP3 is itself able to increase its own transcription *via* p65, we checked for the IMP3 reporter activity in IMP3 overexpression conditions. We see an increased IMP3 promoter dependent luciferase reporter activity in IMP3 overexpression condition in LN229 cells (Figure 4I) and this increase was abrogated by BAY 11-7082 treatment (Figure 4J). These results demonstrate the existence of a probable positive feedback loop between IMP3 and NF-κB pathway in GBM.

### IMP3-p65 cascade also regulates glioma stem-like cells (GSC) maintenance and migration

**Figure 5:**
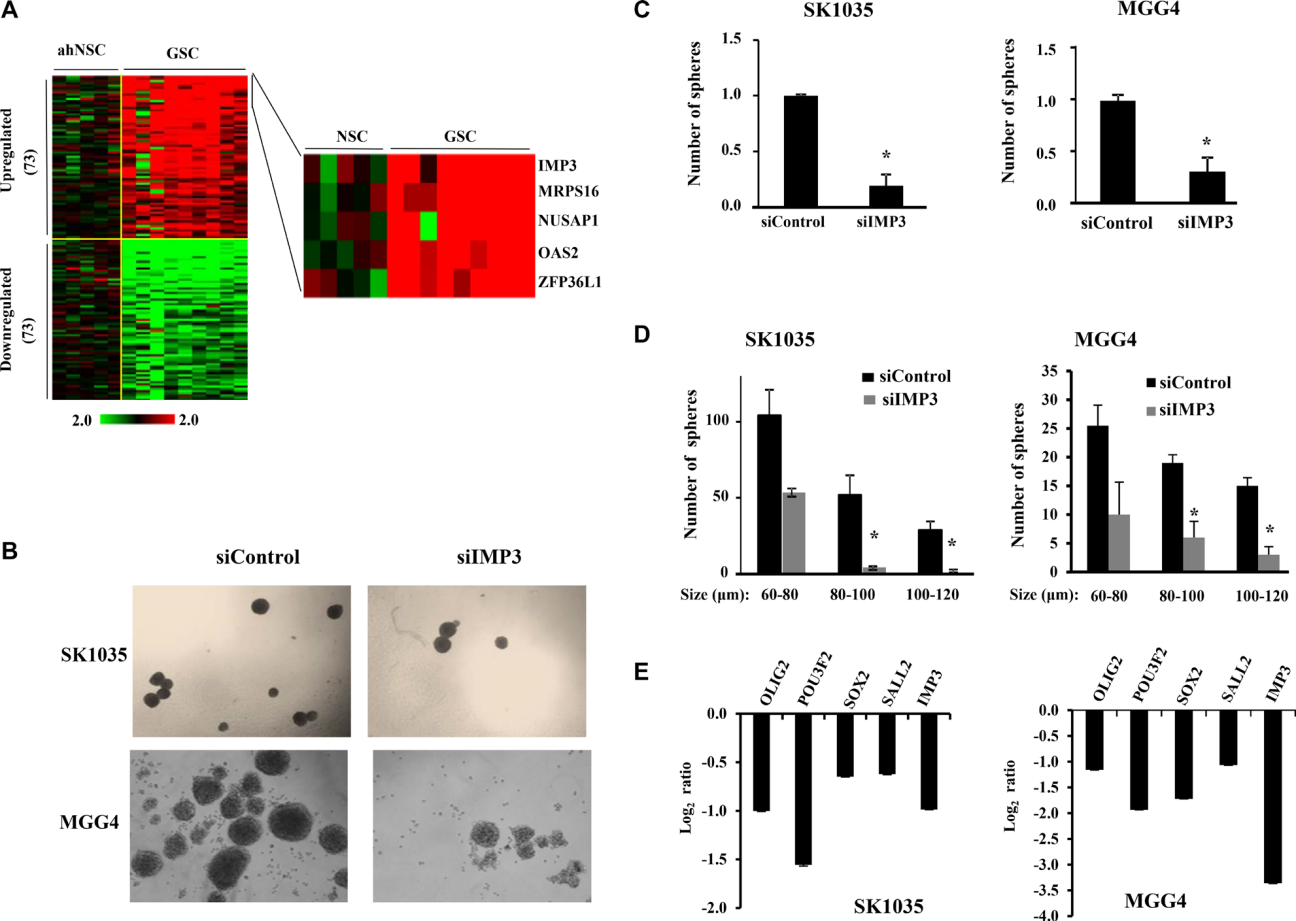
IMP3 expression is associated with GSC maintenance (**A**) Heat map showing significantly differentially expressed RNA binding proteins (73 up-regulated and 73 downregulated) between normal neural stem cells (*n* = 5) and glioma stem like cells (*n* = 8) from GSE31262. Log_2_ ratio values of expression are plotted ranging from −2 (green: downregulated) to +2 (red: upregulated). (**b**) Representative images showing decrease in sphere number and diameter after IMP3 silencing mediated by siRNA for SK1035 and MGG4 GSCs. (**C**) Quantitative representation of the relative decrease in sphere number after IMP3 depletion in GSCs using bar graph. The number of spheres in untreated condition was considered to be 1 and number of spheres in knockdown condition is plotted relative to it. (**D**) Number of spheres falling in different size categories (as per diameter in μm) plotted as bar graphs. Note that in IMP3 knockdown condition there is a general decrease in sphere diameter, but the number of spheres with bigger diameter is affected more severely in both SK1035 and MGG4 GSCs. (**E**) qRT-PCR analysis of transcript levels of known glioma stem-like cell markers upon IMP3 knockdown in SK1035 and MGG4 GSCs. Log_2_ ratio values of the transcript levels are plotted. For all experiments, a student *t-test* was carried to assign statistical difference in observations made in the conditions indicated. A *p* < 0.05 is represented with *p < 0.01 is represented as ** and *p* < 0.001 is represented as ***.

**Figure 6:**
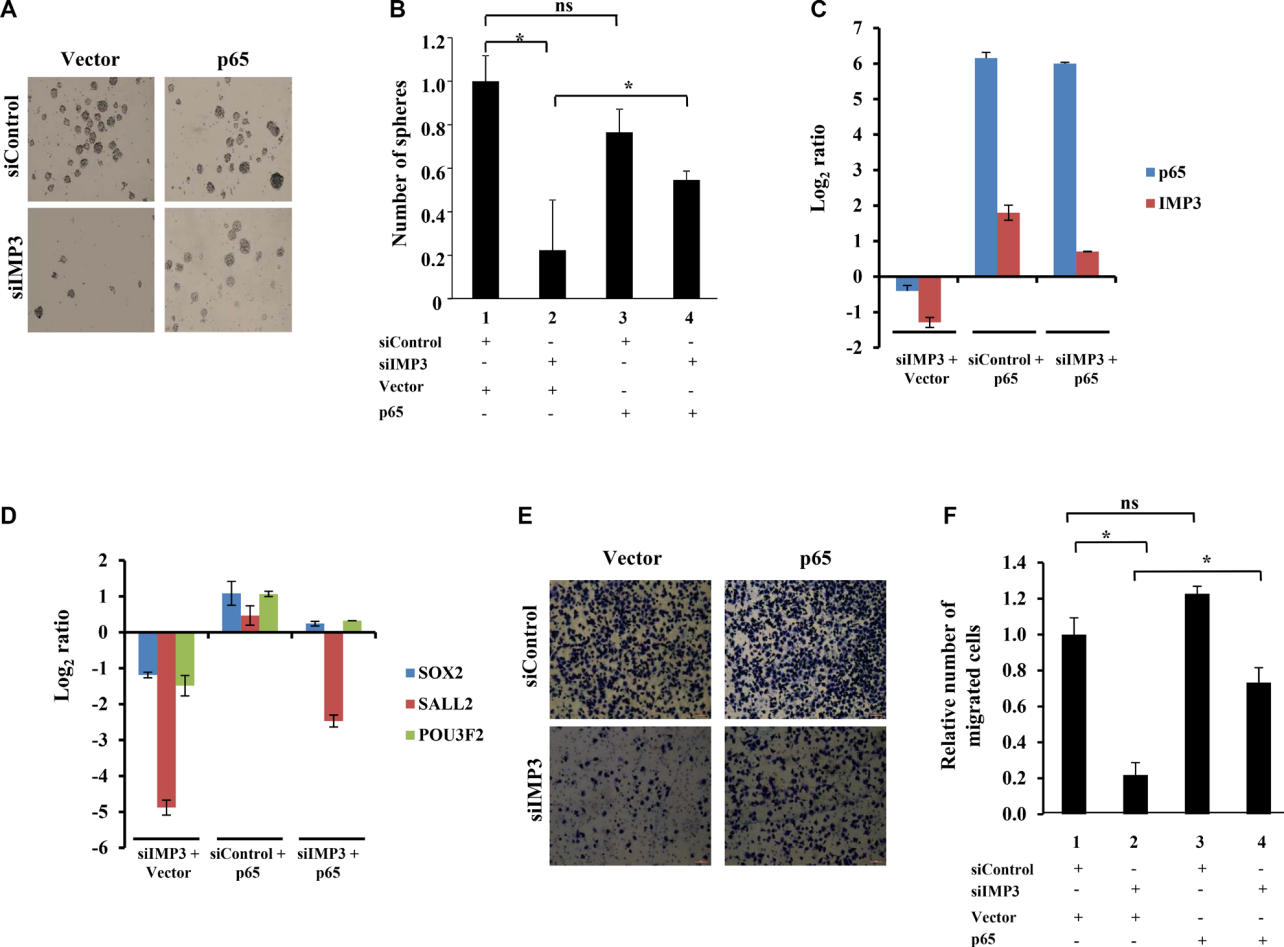
IMP3 regulates GSC maintenance and migration via p65 (**A**) Representative images of SK1035 neurospheres formed in the four mentioned conditions: siControl with control vector transfection, siControl with p65 overexpression vector transfection, siIMP3 with control vector transfection and siIMP3 with p65 overexpression vector transfection. (**B**) Quantification of neurospheres formed in the indicated conditions. (**C**, **D**) Transcript levels of IMP3, p65, SOX2, SALL2 and POU3F2 genes in SK1035 under the mentioned conditions: siControl with control vector transfection, siControl with p65 overexpression vector transfection, siIMP3 with control vector transfection and siIMP3 with p65 overexpression vector transfection. (**E**) Representative micrographs of migrated SK1035 GSCs in the mentioned conditions: siControl with control vector transfection, siControl with p65 overexpression vector transfection, siIMP3 with control vector transfection and siIMP3 with p65 overexpression vector transfection. (**F**) Quantitation of the number of migrated cells is represented as a bar graph. For all experiments, a student *t-test* was carried to assign statistical difference in observations made in the conditions indicated. A *p* < 0.05 is represented with *p < 0.01 is represented as ** and *p* < 0.001 is represented as ***.

Since GSCs show enhanced migratory property and IMP3 promotes migration of glioma cells, we were intrigued if there is any association between IMP3-p65 cascade and GSCs [17, 26–28]. We examined the transcript levels of 1756 RNA binding proteins (RBPs) [29, 30] derived from GSE31262 microarray database, which provides transcript profile of five adult human neural stem cell lines (ahNSC) and nine glioblastoma stem cells lines (GSC) [31]. There were 73 RBPs upregulated and 73 RBPs downregulated in GSC in comparison to ahNSC (Figure 5A). Among the GSC specific upregulated RBPs, we found IMP3 to be the most upregulated (Fold change in GSC over ahNSC = 92.94), suggesting that IMP3 may play an important role in GSC maintenance (Figure 5A; Supplementary Table 2). To confirm the association between IMP3 and GSCs, we carried out Gene Set Enrichment Analysis (GSEA) for the possible enrichment of gene expression profile of CD133+ (a marker for glioma stem-like cells) glioma cells [32], in IMP3 expressing glioma tumors. We found a positive enrichment of upregulated genes in CD133+ glioma cells (CD133+_up) in GBMs with high levels of IMP3 transcripts (IMP3 high) (Supplementary Figure 4A). In contrast, GBM tumors with low levels of IMP3 transcripts (IMP3 low) had a positive enrichment of downregulated genes in CD133+ glioma cells (CD133+_down) (Supplementary Figure 4B). To experimentally validate the importance of IMP3 in GSC maintenance, we tested the effect of IMP3 silencing on the ability of GSCs (SK1035 and MGG4) to grow as neurospheres. IMP3 silencing inhibited GSC neurosphere growth significantly both in terms of number and size (Figure 5B–5D). SK1035 and MGG4 GSCs have been described previously [33, 34] and were grown as neurospheres, which are known to enrich cells with stem-like characteristics. IMP3 silencing also resulted in the downregulation of four glioma reprogramming transcription factors- OLIG2, POU3F2, SOX2 and SALL2 in GSCs (Figure 5E). These results were also reproduced in glioma cell lines, where a diminished neurosphere forming capacity was observed in A172, U87 and U251 cells transfected with siIMP3 (Supplementary Figure 4C and 4D). Next, we tested whether p65 is also a critical downstream mediator of IMP3 in GSC maintenance using SK1035. The inhibitory effect of IMP3 depletion on neurosphere formation was tested in the presence of exogenously expressed p65 transcribed from a 3′UTR-less construct. IMP3 downregulation led to significant decrease in number of neurospheres as observed before (Figure 6A and 6B; compare bar 2 with 1). However, simultaneous exogenous overexpression of p65 rescued the neurosphere formation significantly (Figure 6A and 6B; compare bar 4 with 2). qRT-PCR confirmed the efficient silencing of IMP3 and p65 overexpression (Figure 6C). We also observed an increase in IMP3 transcript in p65 overexpression condition establishing the regulation of IMP3 transcription by p65 in GSCs also (Figure 6C). The spheres formed upon p65 overexpression in IMP3 silenced condition indeed showed increased levels of SOX2, SALL2 and POU3F2 (Figure 6D). Next, we also checked if migration capacity of GSCs is regulated by IMP3. IMP3 knockdown led to decreased migration of SK1035 GSCs (Figure 6E and 6F; compare bar 1 with 2). Further, we also observed an alleviation of reduction in migratory potential with p65 overexpression in IMP3 depleted cells (Figure 6E and 6F; compare bar 2 with 4). Taken together, these results underscore the importance of IMP3-p65 cascade in maintenance of glioma stem-like cells and their migratory capacity.

## DISCUSSION

IMP3 is an oncofetal RBP, implicated in migration, invasion, angiogenesis, cancer stem-like cell maintenance and is also associated with malignant and recurrent tumors [17, 35–41]. IMP3, being an RNA binding protein, has promiscuous binding and a plethora of target transcripts [18, 19]. From the multiple targets, we were interested to identify a global regulator, regulated by IMP3 at the level of translation, which may be playing a crucial role in migration of glioma cells. We focussed our efforts to IMP3 mediated migration as our previously published results in GBM clearly reflect the importance of this molecule in this hallmark of cancer [17]. Our integrated bioinformatics approach identified p65, a transcription factor in NF-κB pathway to be a putative target of IMP3. NF-κB pathway activates a cascade of genes important during development and is also associated with aggressive and resistant tumors [42–44]. In addition, the level of NF-κB activation is directly proportional to tumor aggressiveness [45], and also implicated in cancer stem-like cell maintenance [24, 46–48]. In GBM, NF-κB pathway is constitutively active in a subset of tumors and represents a survival signal sustaining tumor growth [23]. It is also reported to play an important role in migration, invasion, GSC survival and chemoradiotherapy by its ability to activate various important oncogenes, including IL-6, IGFBP-2 and c-myc, all of which are known regulators of migration capacity, glioma stem-like cells and are associated with tumor aggressiveness [49–51]. These reports motivated us to validate p65 as a target for IMP3 and as its downstream mediator in rendering migratory potential to the cells. Our findings in glioma cells with IMP3 overexpression and silencing unequivocally establish IMP3 as a positive modulator of NF-κB signalling by increasing the translation of p65 transcript. These results are in coherence with the observations made by Pei et al., where they have shown that IMP3 activates NF-κB pathway and contributes to migration of renal cell carcinoma cells [52]. Our p65 rescue experiment in IMP3 depleted U138 glioma cells clearly reflects the importance of the molecule downstream to IMP3 in imparting migratory potential to these cells. We also show that IMP3 has a role to play in GSC maintenance and migration. GSCs are known for high migratory potential and therapy resistance [4, 27, 53] thus the association of IMP3 with GSCs makes it a lucrative target for therapy.

Another aspect investigated is the binding of IMP3 to p65 3′UTR. Our results establish a specific binding of IMP3 to three sites on the p65 3′UTR. In luciferase assays with p65 3′UTR, we observed that mutation in site 1 did not significantly alter the enhanced activity in IMP3 overexpression conditions (as observed in wild type 3′UTR construct). Conversely, mutation in either site 2 or site 3 significantly impaired the IMP3 mediated increase in luciferase activity. This signifies the importance of IMP3 binding to these two sites (site 2 and site 3) in mediating its regulation in the cells. Though there may be various reasons behind this observation, one of the scenarios that we hypothesize is that IMP3 may be binding to site 2 and site 3 in a pseudodimeric configuration as proposed for IMP1 (a paralogue of IMP3) to its target [54]. Though the exact mechanism by which IMP3 promotes translation of target transcripts is not known, we hypothesize that it may involve recruitment of auxiliary proteins that may act as translation activators.

**Figure 7:**
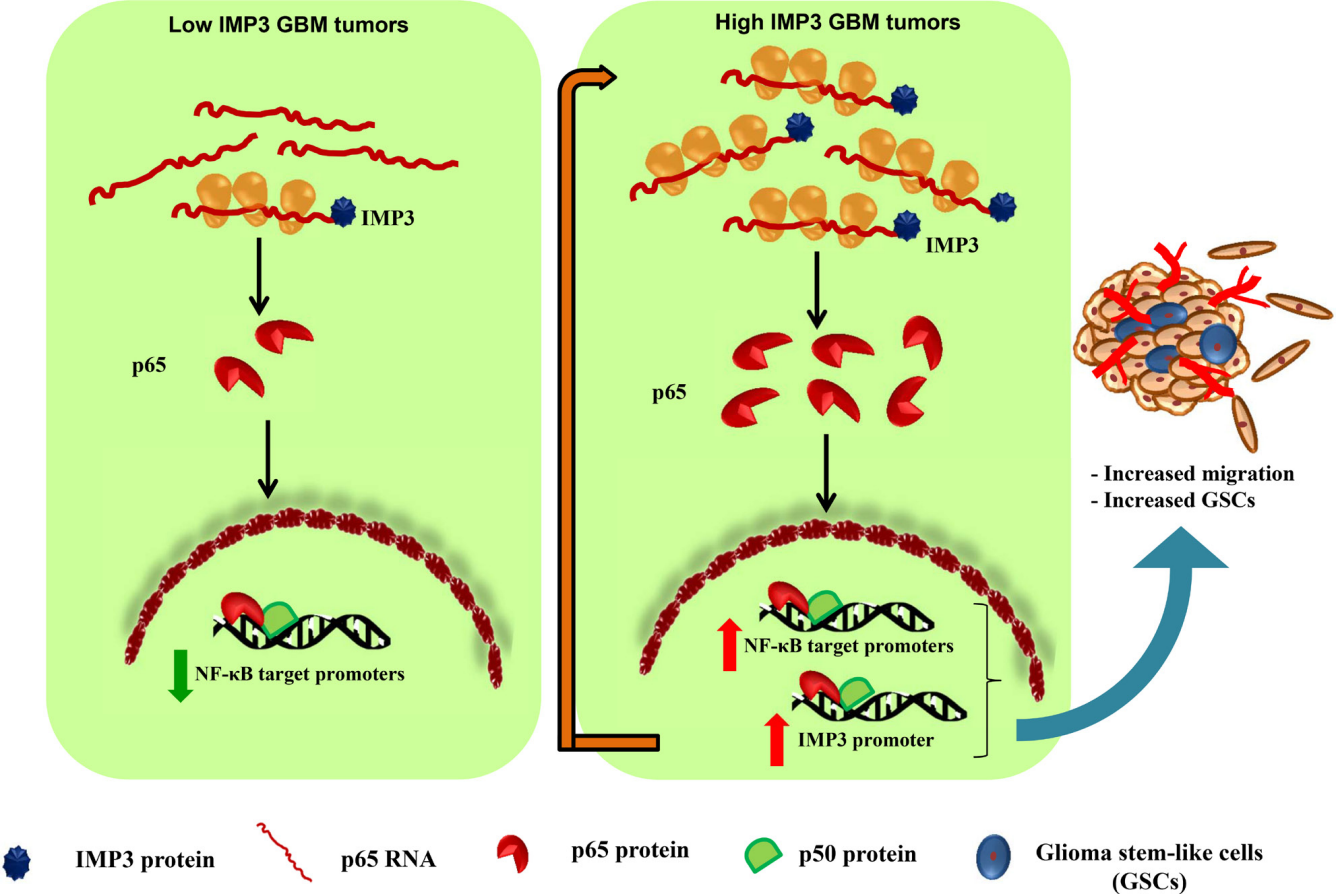
Proposed model of regulation of p65 and IMP3 in GBM tumors Low IMP3 expressing GBM tumors are relatively less invasive in nature. High IMP3 expressing GBM tumors will have high IMP3 protein resulting in enhanced translation of p65 transcript, and thus an increased expression of p65 protein is observed. The activated p65 translocates to the nucleus and induces the expression of NF-κB target genes including several cytokines and oncogenes. IMP3 also acts as another transcriptional target of p65 and the levels of both molecules are regulated in a positive feedback loop (depicted by long red arrow). This contributes in enhanced glioma cell migration and GSC maintenance in high IMP3 expressing GBM tumors.

GBM tumors have high expression of p65 and IMP3 proteins [17, 45]. Our results explicitly establish p65 as a target of IMP3 at the level of translation and also IMP3 as a transcriptional target of p65. In light of the evidence provided, we conclude the existence of a positive feedback loop between IMP3 and p65. The presence of such a positive feedback loop in GBM tumors will further fuel the belligerent state of the tumor. Thus, the disruption of this axis should bring down the aggressiveness of the tumor and its relapse. Taken together, we propose a model wherein IMP3 may enhance the translation of p65 thus contributing to activation of NF-kB pathway in GBM tumors. This may result in transcription of various oncogenes including cytokines and IMP3 itself. Thus our study establishes that the intricate regulation between IMP3 and NF-κB pathway is essential for migration of glioma cells (Figure 7).

The importance of this study relies on the fact that there are limitations in the utilization of NF-κB inhibitors for the management of various cancers. Pre-clinical trials of NF-κB inhibitors have shown promising reduction in tumor burden, but serious concerns about side effects are still raised [55]. These observations thrust upon the importance of alternate strategies for NF-κB pathway inhibition. Molecules targeting IMP3 could be considered for attenuating and disrupting NF-κB thus ensuring pronounced tumor specific inhibition with minimum side effects. Previously published report from our group demonstrated that IMP3 depletion results in enhanced sensitivity to chemotherapy in glioma cell lines [17]. Current study establishes that IMP3 is critical for migration of both differentiated and glioma stem-like cells. Thus, IMP3 depletion may render even the GSCs, which have higher migratory potential and are refractory to the existing therapeutic modalities sensitive to chemotherapy. Further, it has been shown that peptides derived from IMP3 induce immune response of helper T cells and cytotoxic T lymphocytes resulting in better clinical response in esophageal cancer [56, 57]. Hence, IMP3 targeting may prove to be useful as an adjuvant therapy leading to targeting highly infiltrative cells thus improving the median survival, quality of life of patients and reducing the probability of recurrence in a patient.

## MATERIALS AND METHODS

### Culture of adherent cells and glioma stem-like cells

293T, U87 and HCT116 (Sigma); LN229, U138, U343, U251 (from Dr. Abhijit Guha's laboratory), were grown in Dulbecco's modified Eagle Medium (DMEM) supplemented with 10% Foetal Bovine Serum (10% FBS), penicillin (Sigma, U.S.A.), gentamicin (Sigma, U.S.A.) and streptomycin (Sigma, U.S.A.) at 37^°^C in a humidified atmosphere with 5% CO_2_. Primary tumor GSCs were kindly given by Dr. Santosh Kesari (University of California, San Diego) and Dr. Wakimoto H. (Massachusetts General Hospital, Boston). They were cultured in Neurobasal medium (Invitrogen, U.S.A.) supplemented with 3 mmol/L L-Glutamine (Invitrogen, U.S.A.), basic fibroblast growth factor (bFGF; 20 ng/ml, Promega), epidermal growth factor (EGF; 20 ng/ml; Promega), 1X B27 supplement (Invitrogen, U.S.A.), 0.5 × N-2 (Invitrogen, U.S.A.), 2 μg/mL Heparin (Sigma, U.S.A.), penicillin, gentamicin and streptomycin in ultra-low attachment plates (Corning, U.S.A.). SK1035 neurospheres were passaged by mechanical dissociation after days 8–10, while chemical dissociation kit (Catalog# 05707, STEMCELL technologies, U.S.A.) was used for dissociation of MGG4. Fresh medium was supplemented every 2–3 days.

### Human tumor samples

Source of tumor samples are GBM patients operated at National Institute of Mental Health and Neurosciences (NIMHANS) and Sri Sathya Sai Institute of Higher Medical Sciences (SSSIHMS), Bangalore, India. Tissues were bisected and one portion was used for placed in RNA later (Ambion Inc., USA), stored at −70^°^C and used for RNA isolation, while the other half was fixed in 10% buffered neutral formalin, processed for paraffin sections and was used for immunohistochemistry (IHC). The study was approved by the ethics committee of NIMHANS and SSSIHMS (the two clinical centres) and the consent of the patients was obtained as per the Institute Ethical Committee guidelines and approval. For assessing the transcript levels of various genes (IMP3 and RELA) in tumor samples we had used forty six GBM samples.

### Plasmids and other reagents

IMP3 overexpression construct used has been described earlier [17]. IMP3 promoter reporter was procured from Switch Gear genomics (S717055) and sub-cloned into pGL3 Basic vector (Promega, U.S.A.). NF-κB dependent luciferase reporter vector was a kind gift from Dr. Balaji, IISc, India. IκB super-repressor (IκBSR) was kindly provided by Dr. Inder Verma. pAG23 RELA 3′UTR was a gift from David Bartel (Addgene plasmid # 14505).

Control siRNA pool (Catalog # D-001810-10-50) and siRNA pool used against IMP3 (GenbankTM accession number NM_006547; Catalog # L-003976-00-0050) were purchased from GE HealthCare Dharmacon Inc (On-TARGET plus Human siRNA SMART pool). These siRNA against IMP3 contains a pool of 4 siRNAs. The details of these are given in previously published literature [17]. GFP Adenovirus and IMP3 adenovirus used were described earlier in previously published literature [17]. BAY 11-7082 (Calbiochem, U.S.A.) and TNF-α (Cell Signaling Technology, U.S.A.) were also used in this study.

### RNA isolation and real-time quantitative RT-PCR analysis

Total RNA was isolated using TRI reagent (Sigma, U.S.A.) and 2 μg of RNA was reverse transcribed using the High capacity cDNA reverse transcription kit (Life technologies, USA) according to the manufacturer's protocol. qRT-PCR was performed using the ABI PRISM 7900 HT Sequence Detection System (Life technologies, USA). Expression of the genes of interest was analyzed and normalized to 18S rRNA, ATP5G1, AGPAT or RPL35a as internal control genes following the ΔΔCt method [58]. The primer sequences used for p65 and IMP3 are *p65 (forward)*: GAAGAAGAGTCCTTTCAGCG, *p65 (reverse)*: GGGAGGACGTAAAGGGATAG, *IMP3 (forward)*: CACCTCTGCGGCTTGTAAGTC, *IMP3 (reverse)*: CAGCGTCAATTCCTGCAATGG, *POU3F2 (forward)*: TGACGATCTCCACGCAGTAG, *POU3F2 (reverse)*: GGCAGAAAGCTGTCCAAGT, *SOX2 (forward)*: AACCCCAAGATGCACAACTC, *SOX2 (reverse*): GCTTAGCCTCGTCGATGAAC, *SALL2 (forward)*: TAATCTCGGACTGCGAAGG, *SALL2 (reverse)*: TAGAACATGCGTTCTGGTGG, *OLIG2 (forward)*: CCAGAGCCCGATGACCTTTT, *OLIG2 (reverse)*: AGGACGACTTGAAGCCACTG

### Western blotting

Equal amount of lysates (quantified using Bradford reagent) were resolved on SDS-PAGE gels, transferred on PVDF membrane and probed with antibodies against IMP3 (Catalog # HPA002037, Sigma, U.S.A.), actin (Catalog # A3854, Sigma, U.S.A.), PCNA (Catalog # NA-03, Calbiochem, U.S.A.) and p65 (Catalog # 3034S, Cell Signaling Technology, U.S.A.). For cycloheximide chase experiment, cylcoheximide (50 μg/ml) (Sigma, U.S.A.) was added to the cells and the cells were harvested at the indicated time points. The lysates were made and the western blotting was performed. The blots were probed for p65 and the loading control.

### Immunofluorescence staining

Cells were fixed with 4% paraformaldehyde, washed with Tris-buffered saline, and incubated with anti-p65 (Catalog # 8242, Cell Signaling Technology, U.S.A.). Primary antibodies were incubated for 16 h at 4^°^C followed by detection with Alexa 488 anti-rabbit (Invitrogen). Nuclei were stained with DAPI (Sigma) and slides were mounted using anti-fade (Invitrogen). Images were taken with a confocal microscope (Leica, U.S.A.).

### Luciferase reporter assay

IMP3 promoter region was purchased from Switch gear genomics and the promoter region was sub-cloned in pGL3-Basic vector. NF-κB reporter luciferase construct was a kind gift from Dr. K.N. Balaji (IISc., Bangalore India). Luciferase assays were performed using reporter lysis buffer RLB (catalog # E3971, Promega, U.S.A.) and Luciferase Assay reagent (LAR, catalog # E1500, Promega, U.S.A.) as per manufacturer's instructions. Briefly, plasmids were transfected in the cells plated in 12 well plates using Lipofectamine 2000 (a total of 2–4 μg in each well). Cells were harvested after 48 h of transfection and lysates were made. Luciferase readings were recorded using luminometer. β-galactosidase assays or Renilla luciferase readings were performed to normalize the transfection differences. These assays were carried in duplicates.

For 3′UTR luciferase assays, the plasmid was obtained for Addgene. The wild type or mutated 3′UTR plasmids (containing Renilla luciferase) were co-transfected with pCMV-luc (having firefly luciferase gene) and the luciferase assays were performed using dual luciferase assay kit (Catalog # E1910, Promega, U.S.A.).

### Transfection of GSCs, neurosphere assay and sphere diameter measurement

GSCs transfections were carried out in single cell suspension state for both siRNA and plasmids. After 48 h of transfection, the aggregates formed were dissociated into single cells, counted and equal numbers of cells were plated at a density of 4 cells/μl in 24-well or 6-well plate. Number of spheres was counted after 7 days of plating. Again, fresh medium was added to the wells every 2–3 days. Sphere diameter measurements were done with ImageJ software. Number of spheres above the median diameter were calculated and plotted for total number of spheres. These assays were performed in duplicates.

### Migration of glioma cells and GSCs

U138 glioma cells and SK1035 GSCs were transfected with control siRNA or siRNA against IMP3. After 36 h of transfection, cells were plated for p65 overexpression transfection (5 μg in a 35 mm). After 36 h of p65 transfection, cells were made into single cell suspension and 25,000 cells were plated for migration in a Corning^®^ BioCoat^™^ Control Inserts with 8.0 μm PET Membrane (Catalog # 354578, Corning, U.S.A.). Upper chamber contained incomplete medium required for the growth of respective cell lines, while the lower chamber contained medium with 20% serum. Migration of cells was observed after 8 h of incubation in 37^°^C incubator. Cells from the upper side of the chamber were gently removed, while those in the lower side were fixed using chilled methanol (30 min) and stained with 0.1% crystal violet (30 min). Representative images were taken under light microscope and migrated cells were counted. The assay was performed in duplicates.

### IMP3 protein purification

pET42a-IMP3 was obtained as a gift from Jan Christiansen. Untagged protein was purified as mentioned before without any modifications [59].

### *In vitro* RNA transcription and site directed mutagenesis

DNA fragments were obtained by amplifying the site 1, site 2 and site 3 from the pAG23 RELA 3′ UTR plasmid. Amplification of region from each site was done using forward primer having T7 promoter which were then used as template *in vitro* transcription. These transcripts were also labelled using α-^32^P during the reaction. The transcription reactions were carried out using T7 RNA polymerase (Promega, U.S.A.) according to the manufacturer's instructions. Primers used for amplification for templates for *in vitro* transcription are *site 1 (forward)*: TAATACG ACTCACTATAGGGAGATTTTATTGTCAGTATCTG, *site 1 (reverse)*: GTTCCCTACAGAGAAGGGAGCT GACC, *site 2 (forward)*: TAATACGACTCACTATA GGGAGAGGAGGTAAGGCCTTTGAGCC, *site 2 (reverse)*: CGCTGGTGTTAGGCACAGGGACAATGCC, *site 3 (forward)*: TAATACGACTCACTATAGGGAGAG TGCCTAACACCAGCG, *site 3 (reverse)*: GGAACTGAC CAGACCAAACCCCTTCTGG.

Mutation of IMP3 binding consensus in three sites of p65 3′UTR was performed by site directed mutagenesis using the QuickChange^®^ XL Site-directed Mutagenesis Kit (Catalog # 200516) from Stratagene (Agilent Technologies, U.S.A.) following manufacturer's instructions. The primers used for mutagenesis are - *site 1*: GGAGGTGCTTAAGCAGAAGACTTAACTTCTCT GGAAAGGG, *site 2*: GTCTTCCATCATGGATTACTTA CAGCTTAATCAAAATAACGCC and *site 3*: TCT TTCCTTGCTCAACACTGGCTGAAGGAAACCAG. The template used was pAG23 RELA 3′UTR plasmid.

### UV crosslinking of RNA with IMP3 protein

For UV crosslinking, α-^32^P UTP-labelled radioactive probes of the IMP3 binding sites in p65 3′UTR were *in vitro* transcribed. The DNA substrates used were LN229 cDNA, wild type or mutant RELA 3′UTR plasmid (Addgene). The protocol for UV crosslinking was followed as described earlier [60]. Briefly, α-^32^P UTP-labelled RNA probes were incubated with the purified IMP3 protein (1 × = 2.35 pmol) in RNA binding buffer at 30^°^C for RNA-protein complex formation. Protein was quantified using Bradford reagent in spectrophotometer. RNA and protein were subjected to UV crosslinking for 20 minutes. This was followed by RNase A treatment (30 μg) at 37^°^C for 30 min to remove the unbound RNA. Protein-RNA complexes were resolved on SDS-10% PAGE. The gel was subjected to phosphorimaging and analyzed.

### RNA immunoprecipitation (RIP)

Protocol was followed as described earlier [17]. Briefly, LN229 cells were infected with AdGFP or AdIMP3. After 48 h, cells were lysed in lysis buffer containing 100 mM KCl, 5 mM MgCl2, 10 mM HEPES pH = 7.0, 0.05% Nonidet P-40, 1 mM DTT, 100 U/mL RNase inhibitor, and 1X protease inhibitor cocktail. The lysates were spun at 13000 rpm for 30 minutes and protein concentration of supernatants was quantified. Supernatants from each condition (containing equal protein concentration) were incubated with IgG control antibody and anti-IMP3 antibody (AntiIMP3, N19, sc-47893, Santa Cruz) at 4^°^C overnight. BSA blocked Protein-G sepharose beads were added to immobilize the immunocomplexes formed. Washes with lysis buffer containing 10% NP-40 were given, which was followed by Proteinase K and DNase treatment. Finally, RNA was extracted using TRI reagent and precipitated using alcohol. cDNA was made using this RNA as template and used for qRT-PCR for the assessment of the p65 transcript (Primer sequences *forward*: GAAGAAGAGTCCTTTCAGCG, *reverse*: GGGAGGACGTAAAGGGATAG).

### Chromatin immunoprecipitation (ChIP) assays

ChIP procedure followed is detailed in our earlier publication [23]. Briefly, U87 cells were untreated or treated with PBS or TNF-α were processed for ChIP assay. Firstly, to crosslink protein and DNA, cells were incubated with 1% formaldehyde for 10 minutes at 25^°^C. 2.5 M glycine for 5 minutes at room temperature (25^°^C) was used to stop the crosslinking. Cells were washed with cold PBS, lysed using lysis buffer. Chromatin was sonicated (10 sec pulse 10 times, duty cycle 80, power 18) to generate sheared fragments falling in the range of 100bp to 1000bp. Crosslinked chromatin complex was pre-cleared with protein G sepharose beads. p65 antibody (10 μl; Catalog # 8242, Cell Signaling Technology, U.S.A.) or control mouse antibody (IgG) was used to immunoprecipitate the p65 bound DNA fragments. Antibody-p65-DNA complex was captured using protein G sepharose beads and washed. Protein-DNA complexes reverse crosslinked and DNA was eluted. To assay the p65 binding to IMP3 promoter, IMP3 promoter specific primers (forward, 5′- GGCTGCGGTTCCTTTAG-3′ and reverse, 5′- TAGGAGGAGGCGGGATTAGC -3′; amplicon size-115 bp) were used and qRT-PCR was done as described previously. Fold enrichment method was used to calculate the p65 binding to IMP3 promoter in different conditions.

### Bioinformatics analysis

PAR-CLIP data (GSM545209) and knockdown data for IMP3 [19] was downloaded from starBase v2.0 (http://starbase.sysu.edu.cn/) [61] and http://www.mirz.unibas.ch/restricted/clipdata/RESULTS/CLIP_microArray/index.html respectively and analysed. In this analysis, genes having non-significant *p-value* (*p-value* > 0.05) for differential expression mock and IMP3 knockdown conditions and the genes having difference in Log_2_ ratios in range of – 0.57 and 0.57 between these two conditions were taken as unregulated. Stringent cut off (T2C frequency > 0.5) for IMP3 binding to transcripts was used to shortlist genes. Moreover, the list of animal transcription factors was retrieved from AnimalTFDB [20]. Transcription factor list was arranged using the number of binding sites present in the transcript as reflected by T2C positions.

Differential expression of RNA binding proteins in NSC versus GSC was performed using GSE31262. Publically available microarray datasets like TCGA (https://tcga-data.nci.nih.gov) and GSE22866 [62] were used for plotting RNA expression of p65 in IMP3 high and low tumors. The datasets, were stratified into high and low IMP3 expressing tumors with almost equal number of GBM tumor samples: TCGA (high IMP3: *n* = 147; low IMP3: *n* =176) and GSE22866 (high IMP3: *n* = 16; low IMP3: *n* = 12).

MEGA 6 was used to represent the conservation of consensus sequence in IMP3 binding sites at p65 3′UTR. Prediction of p65 binding sites on IMP3 promoter was performed using Physbinder (http://bioit.dmbr.ugent.be/physbinder/index.php) [63].

A computational approach of GSEA [64] was undertaken to evaluate the TCGA Agilent microarray data for the enrichment of genes up regulated and down regulated in GSCs in IMP3 high and low defined tumor groups respectively. Defined gene sets (Yan_UP_IN_CD133_GBM [65], Yan_DOWN_IN_CD133_GBM [65], BEIER_GLIOMA_STEM_CELL_UP [66] and BEIER_GLIOMA_STEM_CELL_DOWN [66]) from the Molecular Signature Database version 3.0 (MSigDB) were used to evaluate whether statistically significant differences existed between the enrichment of gene sets in the two groups of High and Low IMP3 tumors. We acknowledge our use of the gene set enrichment analysis, GSEA software, and Molecular Signature Database (MSigDB) [64] (http://www.broad.mit.edu/gsea/).

### Polysome analysis

The analysis was performed on U138 glioma cells transfected with control siRNA or siRNA against IMP3. Protocol was described earlier [17].

### Histology and immunohistochemistry

GBM tumor sections were processed and stained for p65, IMP3 and Sox-2 expression. 4mm sections were collected on silane coated glass slides. Antigen retrieval was performed by heat treatment in Tris-EDTA buffer (10 mM Tris Base, 1 mM EDTA solution, 0.05% Tween 20, pH 9.0) at 850 W for 5 mins, 600 W for 10 mins and 450 W for 5 mins respectively. After initial processing, the sections were incubated with the mentioned antibodies. Incubation with secondary antibody (MACH-1 Universal HRP-Polymer Detection kit) was followed. 3,3′-Diaminobenzidine (Sigma-Aldrich) was used as chromogenic substrate. A semiquantitative grading scale was used and the intensity of the immunoreactivity was decided as follows: zero (0) if the staining was absent, 1+ for moderate staining and 2+ if it was strong. For IHC, antibodies used were p65 (Catalog # 8242, Cell Signaling Technology, U.S.A.), IMP3 (Catalog # ab109521, U.S.A.) and SOX2 (Catalog # 3579, Cell Signaling Technology, U.S.A.).

## SUPPLEMENTARY MATERIALS FIGURES AND TABLES







## Abbreviations

Abbreviations used are:

GBM: glioblastoma, IMP3- IGF2 mRNA binding protein 3,
RELA: V-Rel Avian Reticuloendotheliosis Viral Oncogene Homolog A,
GSC: Glioma stem-like cells,
PAR-CLIP: photoactivatable ribonucleoside–enhanced crosslinking and immunoprecipitation,
RIP: RNA ImmunoPrecipitation.
