# Supplementary material for: IGF2 mRNA binding protein 3 (IMP3) promotes glioma cell migration by enhancing the translation of RELA/p65
Source: Oncotarget. 2017 Apr 15;8(25):40469–85. doi: 10.18632/oncotarget.17118 (PMC5522290; doi:10.18632/oncotarget.17118)
Supplement: Supplementary file 1 [file oncotarget-08-40469-s001.pdf]

IGF2 mRNA binding protein 3 (IMP3) promotes glioma cell migration by enhancing the translation of RELA/p65

Supplementary Materials

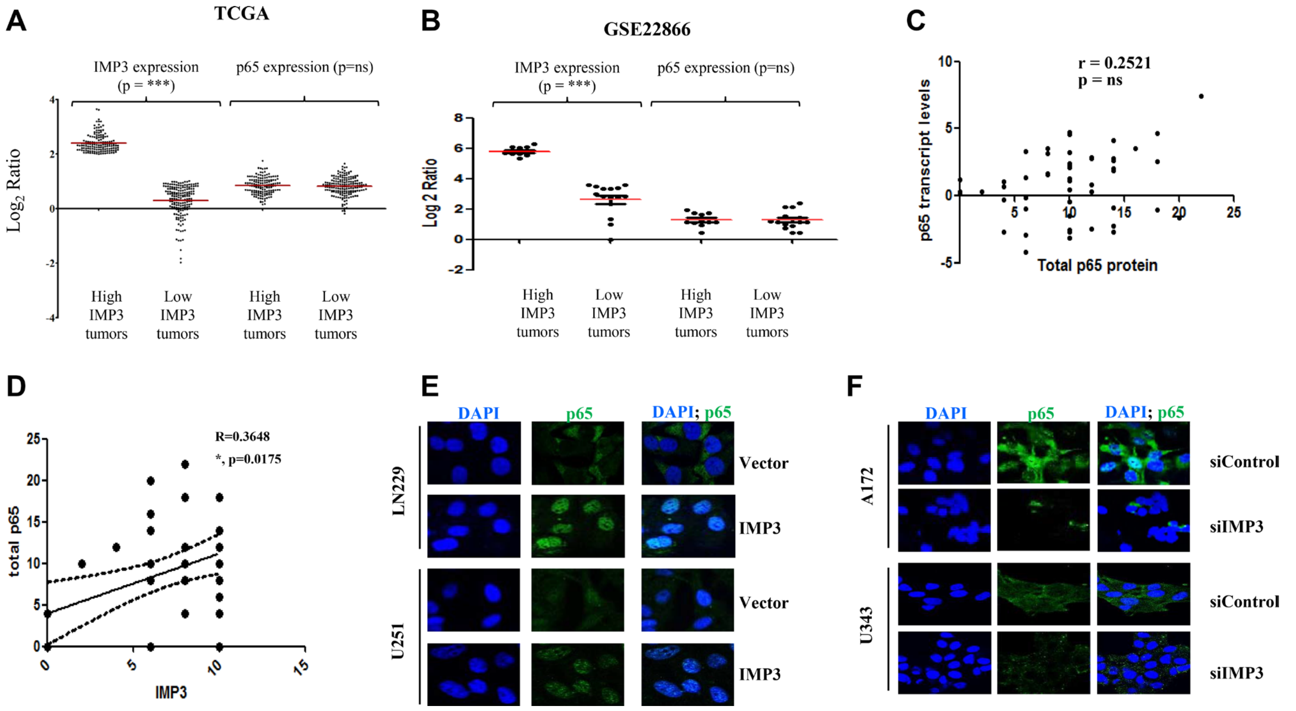

Supplementary Figure 1: (**A**, **B**) Transcript levels of p65 in high- and low- IMP3 expressing GBM tumors in TCGA and GSE22866 datasets. (**C**) Correlation between p65 transcript and p65 protein in GBM tissue samples in our cohort as assessed by qRT-PCR and IHC respectively. (**D**) Correlation between total p65 score and IMP3 protein levels as evaluated by IHC studies on GBM patient samples ($n = 46$) was plotted. (**E**, **F**) Confocal images of p65 stained in glioma cell lines after transfection with IMP3 overexpression construct (E) or with siRNA against IMP3 (F). Note that the cellular localization of p65 gets affected by change in expression levels of IMP3 in the mentioned glioma cell lines.

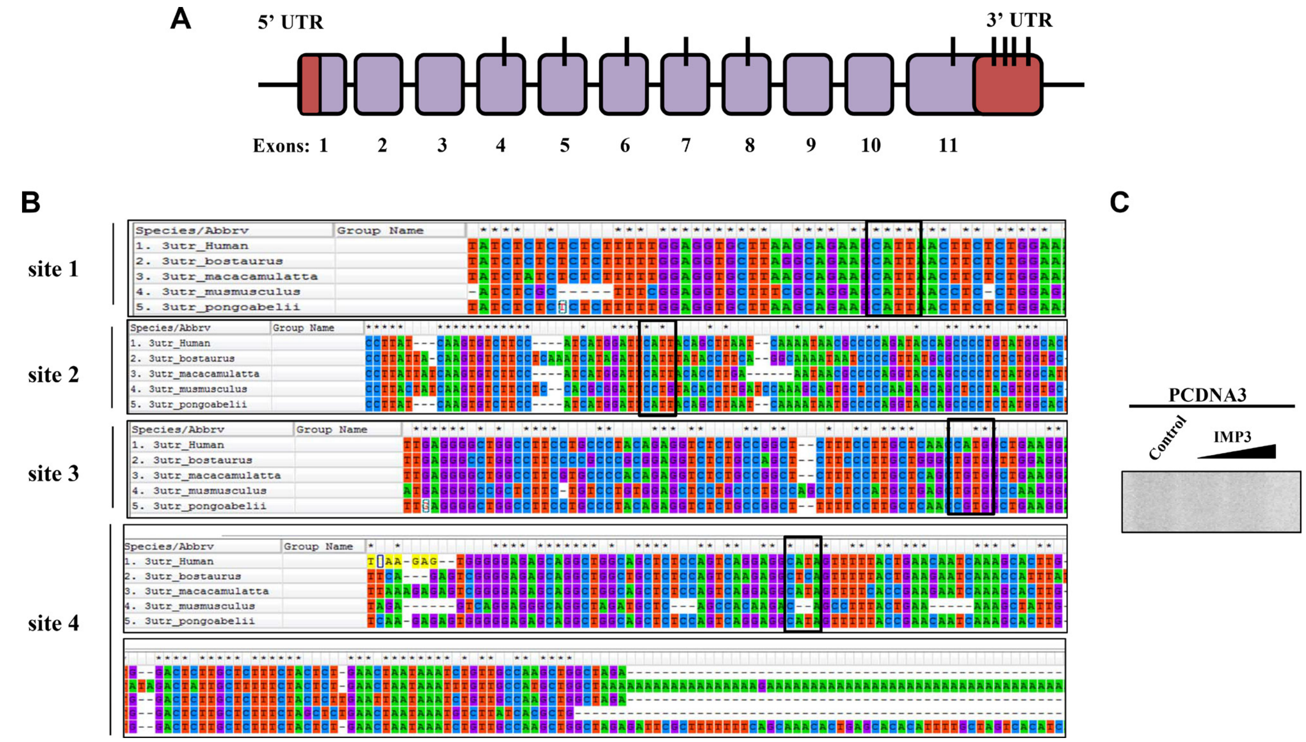

Supplementary Figure 2: (**A**) Schematic representation of putative IMP3 binding sites as predicted by PAR-CLIP. Exons are depicted in purple boxes. Red boxes in the purple boxes indicate the respective UTRs. (**B**) Representation of conservation of four IMP3 binding sites on p65 3′UTR using MEGA 6. The overall conservation of sites goes down from site 1 through site 4, though the conservation of the four nucleotide consensus sites for site 1, site 2, site 3 and site 4 (in black boxes) was 100%, 90%, 85% and 80% respectively. (**C**) Phosphorimage after UV crosslinking of IMP3 purified protein and *in vitro* transcribed non-specific RNA from a region of pCDNA3 (~150 bases).

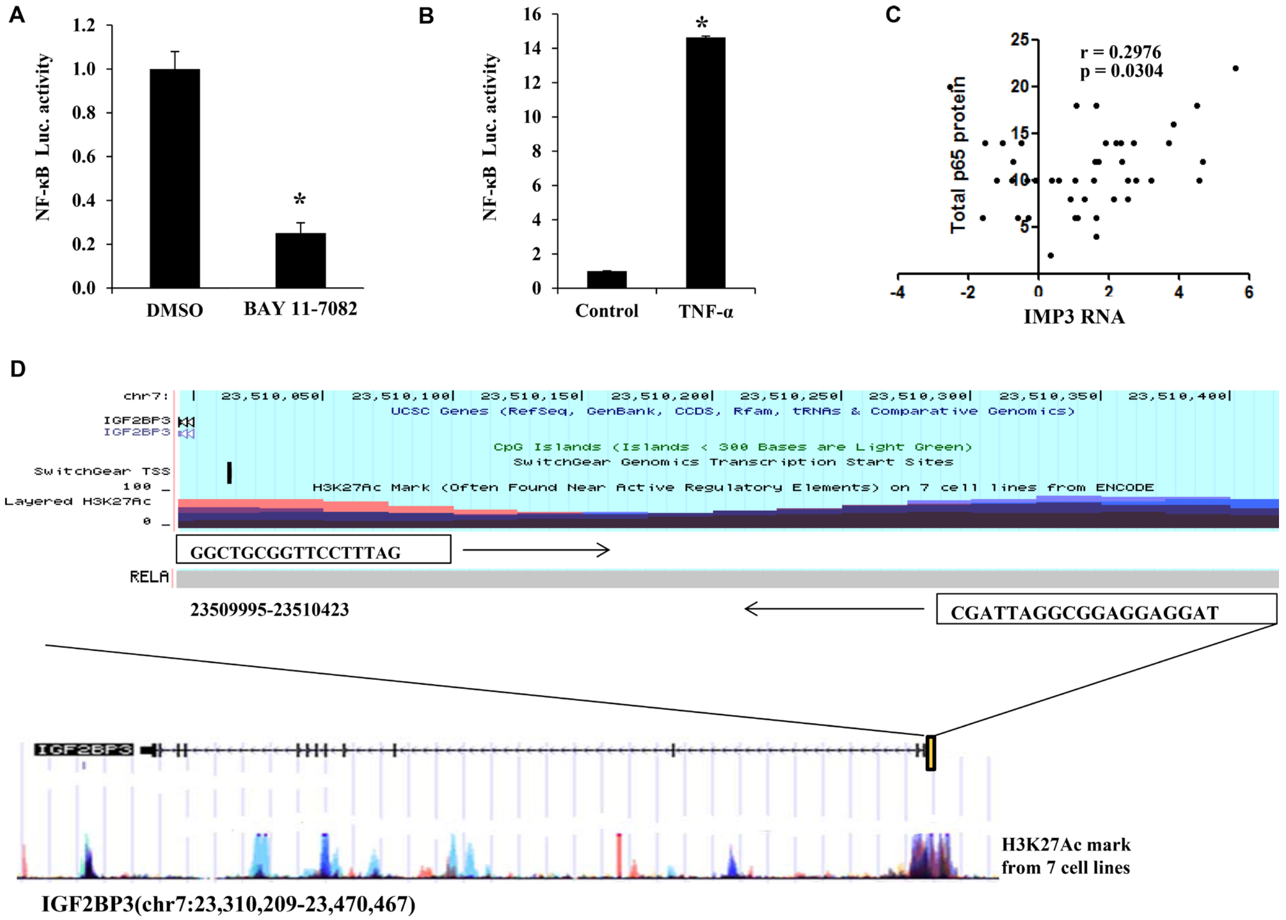

Supplementary Figure 3: (**A**) NF-κB dependent luciferase activity measured in U87 cells treated with BAY 11-7802. (**B**) NF-κB dependent luciferase activity measured in LN229 cells treated with TNF-α (10 ng/ml). (**C**) Correlation of total p65 protein levels (as scored by IHC) and IMP3 RNA levels (as measured by qRT-PCR) in our cohort of GBM patient samples. (**D**) Representation of genomic location of IMP3 and region used for amplification in ChIP assays is shown as in UCSC genome browser. The H3K27 acetylation of the region as found in 7 cell lines is also depicted. RELA (p65) was found to be bound to this region as per the deposited ChIP-seq data with UCSC.

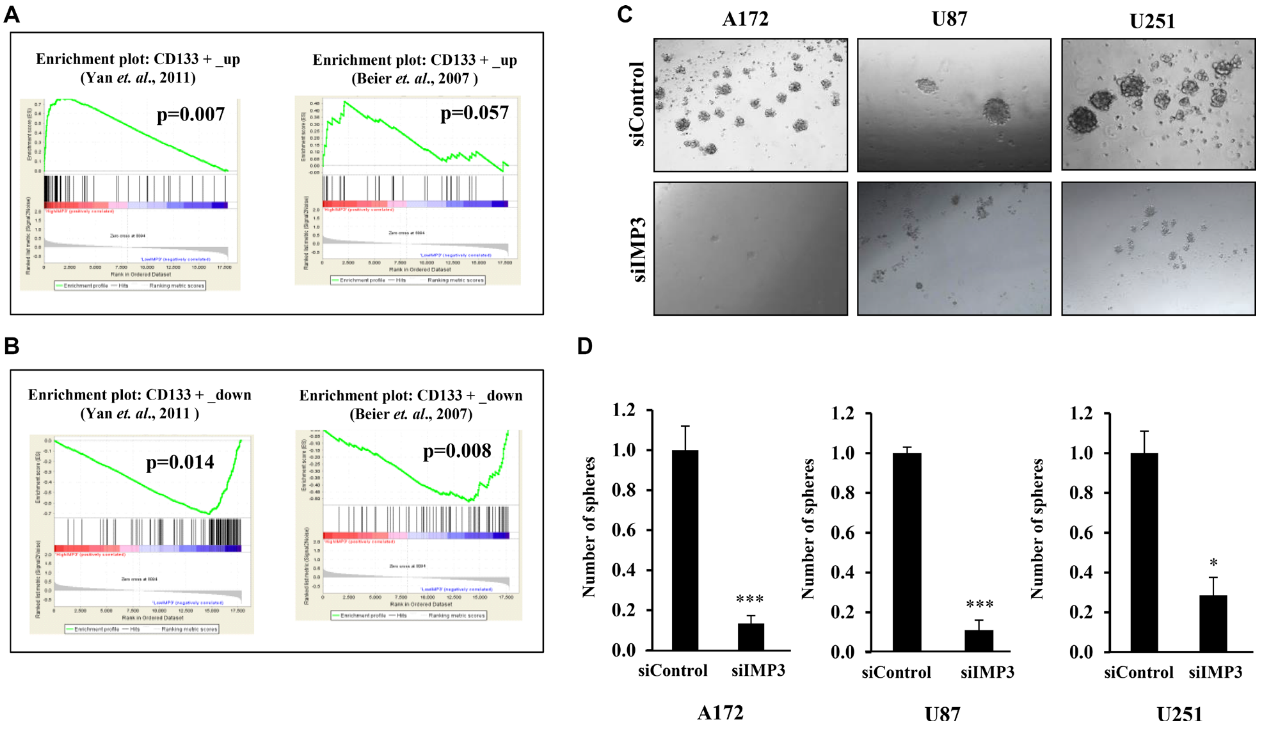

Supplementary Figure 4: (**A**) Gene Set Enrichment Analysis (GSEA) plots for CD133+_up signature genes [72, 73] in IMP3 high tumors (from TCGA). (**B**) GSEA plots for CD133_down signature genes [72, 73] in IMP3 low tumors (from TCGA). (**C**) Representative micrographs of spheres formed from cell lines (A172, U87 and U251) transfected with control siRNA or siRNA against IMP3. (**D**) Relative umber of spheres formed in each condition in all cell lines as in (C) was represented as bar graphs.

Supplementary Table 1: Expression values (in Low IMP3 and High IMP3 tumors from TCGA data) and number of IMP3 binding sites (from GSM545209) for transcription factors found to be unregulated at the transcript level upon silencing IMP3 (from GSE21575). See Supplementary_Table_1

Supplementary Table 2: Differentially expressed RNA binding proteins in GSC and ahNSC in GSE31262. See Supplementary_Table_2
